# A Devasting Course of an Iliopsoas Muscle Abscess Subsequently Leading to Septic Shock, Septic Hip Arthritis, and Extended Gluteal Soft Tissue Necroses in an Elderly Immunocompromised Patient with Multiple Carcinomas: A Case Report and Brief Review of Literature

**DOI:** 10.2174/1874325001812010180

**Published:** 2018-05-31

**Authors:** Ingo Schmidt

**Affiliations:** Med. Versorgungszentrum Bad Salzungen GmbH (Betriebsstätte Wutha-Farnroda), Lindigallee 3, 36433 Bad Salzungen, Germany

**Keywords:** Iliopsoas muscle abscess, Septic shock, Septic hip arthritis, Soft tissue defect, Girdlestone resection-arthroplasty, Coverage, Biceps femoris muscle turnover flap

## Abstract

**Background:**

A devasting course of Iliopsoas Muscle (IPM) abscess remains a challenging therapeutic problem.

**Methods:**

A 69-year-old polymorbid male had a history of multiple carcinomas and presented with advanced stage of septic shock due to a right IPM abscess which communicated with the right hip joint and subsequently led to septic hip arthritis accompanied with post-infectious right gluteal deep soft tissue necroses. Management of surgical treatment included abscess revision, coverage with the use of Long Head Biceps Femoris Muscle (LHBFM) 180° turnover flap, and creating a Girdlestone resection-arthroplasty.

**Results:**

After a duration of patient's hospitalization of six months that included the necessity of artificial respiration over two months accompanied with in summary 18 required surgical procedures, the patient could be recovered successfully regarding his polymorbidity and his low-demand claims in activities of daily living with his Girdlestone resection-arthroplasty.

**Conclusion:**

Recovery of immunocompromised patients with those life-threatening situations can only be achieved by an interdisciplinary management. The LHBFM 180° turnover flap can be useful for filling off post-infectious deep soft tissue cavities communicating with the hip joint. The definitive Girdlestone resection-arthroplasty for treatment of septic hip arthritis is the method of choice for mobilization of elderly polymorbid patients with low demand claims in their activities of daily living.

## 1 INTRODUCTION

Iliopsoas Muscle (IPM) abscess is a rare condition with varying symptomology and etiology and remains a challenging diagnostic and therapeutic problem. Hence, this condition is often primarily overlooked potentially leading to septic shock in up to 20% of cases accompanied with a mortality rate ranging from 5% to 25% with a marked increase of mortality in immunocompromised patients older than 65 years and multiple comorbidities, and untreated cases have a mortality rate of nearly 100% [1-7]. Due to the immediate anatomical relationship of the IPM to the trochanteric bursa which communicates occasionally with the hip joint, the dreaded septic hip arthritis (*i.e.* hip joint epyema) as well as severe infectious-related soft tissue damages around the hip joint can occur.

We present one dramatic course of an elderly immunocompromised patient in whom the IPM abscess, associated with septic shock, hip joint empyema, and deep gluteal abscess has developed by primary etiology or as a secondary late complication either of right nephrectomy 14 years previously and followed by required multiple revision surgery due to retroperitonal abscess formations, or radical prostatectomy 7 years previously, or rectosigmoid colon resection 1 year previously. For treatment management of this life-threatening condition, an interdisciplinary approach is absolutely required. A short review of the literature on the main topics will highlight this publication as well.

## 2 CASE PRESENTATION

**Fig. (1):**
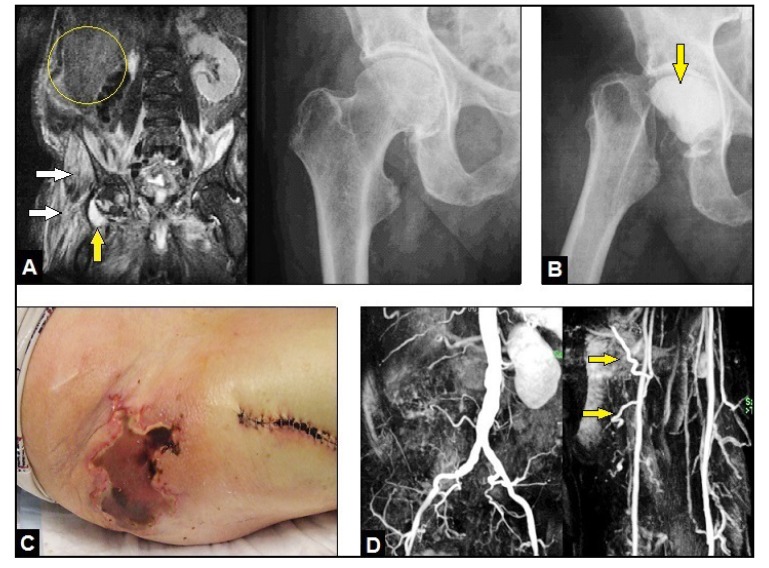
**: (A)** Initial MRI demonstrating right IPM (white arrows) and fluid in right hip joint related to septic hip arthritis (yellow arrow) whereas in radiograph of the right hip there were no signs of infection-related osteolyses at this time. Note the formerly performed right nephrectomy (yellow circle). **(B)** Radiograph of the right hip showing performed GRA with the temporary insertion of a palacos cement spacer containing vancomycin (arrow) 1 week after first presentation. **(C)** Clinical photograph 2 weeks after first presentation showing right deep gluteal pressure-related soft tissue necroses due to MRSA infection. **(D)** MR angiography 4 months after first presentation demonstrating uneventful arterial blood flow through the aorta, both EIAs, both DFA's, and through both FCA's right (yellow arrows).

A 69-year-old male with an advanced stage of septic shock (C-reactive protein 433,2 mg/l, leucocytes 24,7 19 Gpt/l, procalcitonin 2,5 ng/ml) accompanied with an acute respiratory distress syndrome and positive blood culture for Methicillin-resistant Staphylococcus Aureus (MRSA) was assigned to us from another institution. There was a known history of 4 malignancies: a right renal cell carcinoma treated by open nephrectomy (R0) 14 years previously (Fig. **1A**) and followed by required multiple revision surgery due to retroperitonal abscess formations, a prostate carcinoma treated by open radical prostatectomy accompanied with definitive suprapubic urine diversion (R0) 7 years previously, a rectosigmoid carcionoma treated by the open Quenu - procedure resulting with an endstanding anus praeter (R0) and followed by neoadjuvant radio-chemotherapy 1 year previously, and a squamous cell carcinoma at the left thigh treated by required twice excisions (primarily R1, secondarily R0) 6 months previously. Additionally, the patient had a history of hypertensive cardial disorder and diabetes mellitus. As cause for septic shock, Magnetic Resonance Imaging (MRI) revealed an extended right IPM abscess with its penetration into the hip joint, and radiographically, there were no signs of any osteolysis in the femoral head (Fig. **1A**). With the use of MRI, a septic spondylitis / spondylodiscitis could be excluded.

Initially, the patient was stabilized in our intensive care unit, intravenous application of vancomycin was started, and surgical abscess revision through a ventral abdominal incision was done by the visceral surgeon (local drains were placed additionally). After 1 week, the septic serum parameters did not decrease, hence, the excision of the right femoral head was performed by us creating a Girdlestone Resection-arthroplasty (GRA) accompanied with a temporary insertion of a palacos cement spacer containing vancomycin through a lateral incision at the thigh (Fig. **1B**). Histological examination revealed acute osteomyelitis of the femoral head. After that, the septic serum parameters did not decrease markedly again. Another 1 week later, a massive right gluteal skin necrosis occurred pressure-related by a deep gluteal soft tissue abscess formations with positive microbiology for MRSA again according to the initial positive blood culture, and first now in addition to Multidrug-resistant Pseudomonas aeruginosa (MRPA) (Fig. **1C**) that required 15 radical surgical debridements accompanied with negative-pressure vacuum assisted (VAC) therapies combined with further intravenous application of antibiotics.

**Fig. (2):**
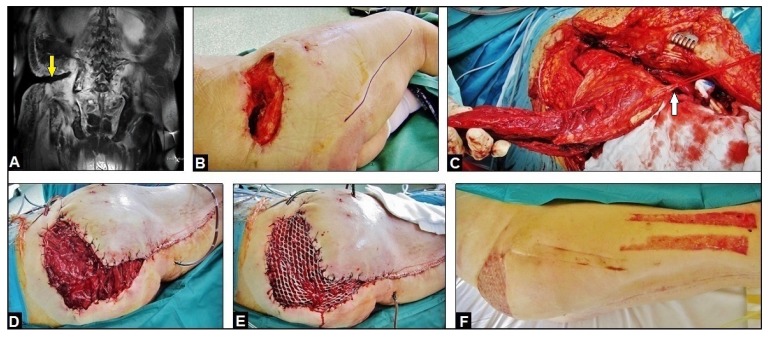
**: (A)** Coronar MRI 4 months after first presentation demonstrating right deep soft tissue gluteal cavity (yellow arrow). **(B)** Clinical photograph 4 months after first presentation showing the right gluteal extended soft tissue defect with it deep cavity which communicates with the sacral/iliac bones and the GRA. The blue line demonstrating planning of surgical incision at the posterior aspect of the right thigh. **(C)** Intraoperative photograph showing the detached LHBFM at its distal insertion at the right fibular head and before it detachement at its proximal origin at the right ischial tuberosity. Note the carefully dissected and obtained major proximal perforator for the LHBFM (white arrow). **(D)** Intraoperative photograph showing transposition of the right LHBFM 180° turnover flap into the right deep gluteal cavity after incision of the adjacent skin bridge. **(E)** Intraoperative photograph showing primary coverage of the right LHBFM 180° turnover flap by splitted skin grafts from the right thigh. **(F)** Clinical photograph 4 weeks after coverage showing uneventful wound healing.

**Fig. (3):**
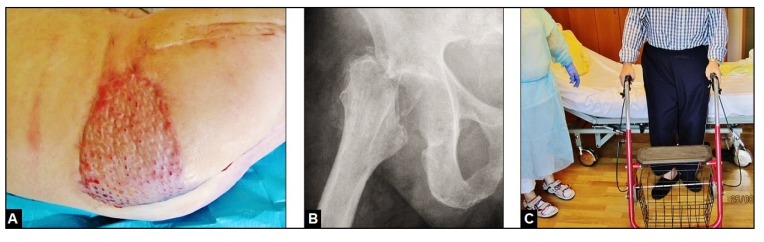
**: (A)** Clinical photograph 8 weeks after coverage (6 months after first presentation) showing uneventful wound healing. **(B)** Radiograph of the right hip 6 months after first presentation demonstrating definitive GRA after the 8 weeks previously removed palacos cement spacer. **(C)** Clinical photograph 6 months after first presentation showing mobilization of the patient with his right GRA by using walking aid. At this time, the required rise off his right shoe was measured with 5 cm by us, and it was manufactured by an orthopaedic shoemaker at home.

Four months after initial presentation of the patient in our hospital, all septic serum parameters were normalized, and the local infection could be controlled. However, the necessity of the multiple soft tissue debridements led inevitably to a large and deep extended soft tissue cavity with communication into the GRA, and to the sacral and iliac bones (Fig. **2A** and **B**). Thus, for filling off the cavity the use of the proximally pedicled Long Head Biceps Femoris Muscle (LHBFM) 180° turnover flap was detected by us. For this purpose, preoperative MRI angiography showed regular perfusion of the External Iliac Artery (EIA), the Deep Femoral Artery (DFA), and both Femoral Circumflex Arteries (FCAs) (Fig. **1D**). The operation was started with a longitudinal incision along the ischial tuberosity - to - fibular head line at the dorsal aspect of the right thigh (Fig. **2B**). All visible perforators from the DFA, popliteal vessel, and sciatic nerve were carefully dissected. Then, all dissected perforators distal to the major proximal perforator were temporarily clipped. After that, the LHBFM showed sufficient perfusion, and so, by preserving the major proximal perforator all distal perforators could be definitively ligated and transected, and the LHBFM was detached from its insertion at the fibular head (Fig. **2C**). Then, the LHBFM was detached at its origin (ischial tuberosity), rotated 180° counter-clockwise at its pivot point (major proximal perforator), and after incision of the adjacent skin bridge positioned into the cavity combined with removal of the palacos cement spacer (Fig. **2D**). The donor site could close completely, and the relocated LBBFM was primarily covered by split skin grafts from the right thigh (Fig. **2E**). Five drains were placed (two into the donor site and three into the cavity under the relocated LHBFM), 17 days after surgery the wound secretion came to standstill and all drains could be removed. Wound healing was uneventful (Figs. **2F** and **3A**).

After a duration of patient's hospitalization of 6 months that included the necessity of artificial respiration over 2 months accompanied with in summary 18 required surgical procedures, the patient could be recovered successfully with walking aid (Figs. **3B** and **3C**) regarding his polymorbidity and his low-demand claims in activities of daily living with his GRA that required additionally a rise of his right shoe with 5 cm. At this time, the patient rated his pain with moderate. A secondary conversion of the GRA to a total hip arthroplasty (THA) was not recommended by us and the patient declined it as well. After that, the patient did not return our hospital again.

## 3 DISCUSSION

Psoas and iliacus muscle originate separately from each other from the bodies of all five lumbar vertebrae and from the superior portion of the iliac fossa, then they extend down through the retroperitoneal space and further beneath the inguinal ligament, pass the hip joint in contact with the trochanteric bursa which communicates occasionally with hip joint, and insert with now only one common (iliopsoas) tendon at the lesser trochanter of the proximal femur. IPM abscess is a rare condition with an incidence of 0,4/100.000 inhabitants reported from the United Kingdom in 1987, but a further incidental increase up to 12/100.000 inhabitants is observed since 1992 that is probably based on an increased disease awareness, development in diagnostic approaches, and an increased number of systemic diseases and malignancies [8, 9].

Regarding the etiology, IPM abscess is classified into primary and secondary origins. Primary etiology makes up approximately 30% of cases, it is more common in children where it can be mistaken for septic hip arthritis, and geographical differences were found as follows: whereas the incidence of primary IPM abscess in Europe is observed in 18,7% of all cases only, the incidence of primary IPM abscess in Asia and Africa accounts up to 90% of all cases [1-4, 10]. Secondary IPM abscess can occur by retrocecal appendicitis, ulcerative colitis, pyelonephritis, inflammatory bowel diseases such as the Crohn's disease, complications after colorectal and urogenital surgery, epidural abscess, non-specific or specific (tuberculosis) vertebral osteomyelitis / spondylitis / spondylodiscitis, pelvic inflammatory disease, local trauma with or without hematoma, septic hip arthritis, and risk factors are immune deficiency such as observed in patients with the human immune deficiency virus syndrome or with malignancies of retroperitonal organs with or without its spontaneous rupture [1-19]. With regard to the anamnestic features in our immunocompromised patient, it could be suggested that the IPM abscess has developed as a secondary late complication after the 1 year previously performed open Quenu – procedure; colorectal surgery due to cancer can be associated with this complication in 32% of all cases [20]. However, it cannot be safely excluded by us that it was probably caused as well by recurrence or exacerbation of IPM abscess after the performed open right nephrectomy with required revision 14 years previously, or as a late postoperative complication after the open radical prostatectomy 7 years previously, or as primary origin as well.

The classic triad of symptoms in patients could have an IPM abscess, first described in 1881 by Mynter [21], are back pain, limp and fever. However, the main reason for initial misdiagnosis is that only in 30% of cases this classic triad actually exists, many of patients report only non-specific single symptoms such as malaise, low grade pyrexia, abdominal or flank discomfort, or pain on movement of the hip which can be fixed in a flexed and externally rotated position [2, 22-24]. Microbiology of primary IPM abscess reveals mostly bacterial load with SA (42,9%) followed by Escherichia coli (EC, 14,3%); whereas in cases of secondary IPM abscess SA with 35,2% followed by Mycobacterium tuberculosis with 17,7% can indicate skeletal origin, in gastrointestinal origin were mostly found EC with 42,1% followed by Bacteroides spp (BS) with 26,3% and in 15,8% each Enterococcus faecalis (EF) / Peptostreptococcus spp, and in urinary origin were mostly found EC with 61,5% followed each by EF / BS with 15,4% [1]. Noted that in secondary IPM abscess caused by gastrointestinal origin SA usually is not observed [1], and so the etiology of IPM abscess with our patient could also be caused by primary origin in the presence of his high-risk factor of pronounced immune deficiency. The secondarily observed presentation of wound infection with MRPA with our patient is a concern, and it is to be considered as complication regarding the necessity of his hospitalization in an intensive care unit [25].

Treatment options for IPM abscess include the intravenous application of antibiotics alone and/or in combination with minimal or open surgical procedures such as percutaneous computerized tomography-guided transabdominal or transperineal catheter drainage or laparotomy. However, none of all procedures are able to provide recurrence in every instance. The overall recurrence rate is reported to be 19% [20]. The success rate for antibiotics alone is reported to be 78% for IPM abscesses smaller than 6 cm, for percutaneous drainage alone 40% and nearly 100% for initial exploratory surgery and drainage in cases with IPM abscesses larger than 6 cm that were found in 39% of cases, and the overall mortality is 5% in patients with a mean age of 53 years [1, 2, 26].

Generally, when septic joint arthritis or osteomyelitis with affection of surrounding soft tissue is present then there is no alternative in surgical treatment for the three-stage management: first, multiple radical bony and soft tissue debridements with or without temporary placement of a cement palacos spacer or polymethyl methacrylate beads containing antibiotics and followed by VAC therapies; second: Coverage of soft tissue defect; and third: Bony and joint reconstruction involving partial or total joint replacement or definitive resection arthroplasty or arthrodesis, but unfortunately limb amputation is not always avoidable in single cases [27-32]. VAC therapy before coverage provides a sterile and controlled environment that can lessen the duration of wound healing, promotes better capillary circulation, and decreases the bacterial load [33]. For the hip joint, Girdlestone first described the removal of the femoral head (*i.e.* resection-arthroplasty) creating a pseudarthrosis in the treatment of tuberculosis [34]. Despite a secondary conversion of the GRA to a THA is possible [35], this salvage procedure is unchanged a reliable and definitive treatment option for elderly patients with low demand claims in their activities of daily living such as in our presented case especially with his MRSA infection. Using this procedure, healing of bony infection without MRSA is attained in 80% to 100% of patients, and worsened when MRSA infection was initially present, hence, for those patients who have additionally immune deficiency and high anaesthetic and operative risks a secondary conversion to THA is not recommended [36, 37]. After surgery, a rise of the patients shoes with a mean of 3,5 cm ranging from 3 cm to 6 cm is needed for their mobilization with a walking aid [38]. However, not all of the patients are satisfied with their GRA. Persistent severe pain is observed in 16% to 33% of patients, and up to 45% of geriatric patients are unable to walk [37].

With our patient, for filling off the large and deep infection-related right gluteal soft tissue cavity which communicated with the sacral/iliac bones and the GRA the use of a large, robust and bulky muscle flap was without alternative. Local pedicled muscle flaps, first reported for treatment of chronic osteomyelitis in 1946 by Stark [39], have proven to be as one useful and reliable surgical option for coverage of soft tissue defects with or without exposure of bradytrophic tissues such as bones and tendons, osteosynthesis material, and joint prostheses [29, 40-43]. Muscle flaps promotes better capillary circulation and decreases the bacterial load, thus, muscle flaps are not to be considered as contraindication when superficial bacterial wound contamination in the absence of manifest and/or deep infection is present. Furthermore, the use of muscle flaps does not require microsurgical expertise, and the loss of function is mostly well compensated by the other agonistic muscles. However, muscle flaps are not free of any problems and complications. Neale *et al*. [44] reported on major or minor complication at the lower extremity in 32% of cases of a total of 95 muscle flaps, and they agreed that the causes were mainly technical errors, inadequate debridement, use of diseased or traumatized muscle, and unrealistic objectives. For treatment of recalcitrant hip joint infection the interposition of the vastus lateralis and/ or rectus femoris muscle has proven to be useful and reliable [45-47]. But to our experience, both the vastus lateralis and rectus femoris muscle flaps from the lateral and anterior aspects of the thigh are too short for filling off a coexistent deep gluteal soft tissue cavity which communicates with the iliac and sacral bones. Utilizing the proximally pedicled LHBFM 180° turnover flap seems to be the more better option for those conditions.

The LHBFM is one of the harmstring muscle group, it lies on the posterior side of the thigh, and it is classified into perfusion type II according to the classification by Mathes and Nahai [49]. Currently, it is not clearly understood whether the major proximal perforators from the DFA and FCA are able to ensure the perfusion of the entire muscle alone [49], or each deep muscular perforator supplies only an isolated segment [50]. A newer anatomical study has shown that the majority of perforator arteries in 94% followed an intramuscular route, the main number of perforators are located in the second and third quarter (including from the sciatic nerve), followed by the first (proximal) quarter, and no perforators were found in the fourth (distal) quarter of the LHBFM [51]. The use of the LHBFM as myocutaneous advancement or V-Y advancement flap has proven to be a suitable and reliable option for coverage of ischial or sacral pressure ulcers [52, 53], but it has a limited upward mobility up to 10 cm only. Thus, the use of the proximally pedicled LHBFM flap in a 180° turnover manner, such as previously described by Bertheuil *et al*. [54] and Demirseren *et al*. [55], which provides a much more upward mobility for filling off extended and deep soft tissue cavities was detected by us. The most common early postoperative complication of this procedure is hematoma and seroma potentially leading to suture dehiscence if the drains were removed before 8 days [54, 55]. Obtaining at least two distal perforators, the LHBFM can also be used as 180° turnover flap for coverage of wounds around the knee joint [51, 56]. The loss of function after separation of LHBFM is sufficiently remedied by the other ischio-crural muscles provided that they are not damaged and there is also no preexistent lateral instability in knee joint; hence, the possible postoperative loss of function and lateral knee laxity would be of relevance for athletes [57-60].

## CONCLUSION

We present a devasting course of an IPM abscess with septic shock and positive microbiology for MRSA that resulted additionally in a septic hip arthritis and a gluteal deep soft tissue defect in an elderly immunocompromised patient. Regarding the specific anamnestic and clinical features with our patient it cannot be safely judged by us if there was a primary or secondary etiology of this life-threatening condition. The patient could be successfully recovered and mobilized with walking aid by surgical revision of IPM abscess, by creating a definitive GRA, and by coverage of gluteal soft tissue defect which communicated with the sacral/iliac bones and the GRA utilizing a proximally pedicled LHBFM 180° turnover flap after multiple debridements within a required long and cost-intensive duration of hospitalization over 6 months. It must be noted that such a devasting condition can only be managed successfully with an interdisciplinary approach between anesthetists, internists, radiologists, visceral surgeons, orthopaedic surgeons, and plastic surgeons if the orthopaedic or visceral surgeons do not have experience in coverage of wounds utilizing various flaps. The patient's course was complicated additionally in the meantime by a secondary MRPA wound infection during the required hospitalization in our intensive care unit. A conversion of the GRA to a THA in such a high-risk patient with immune deficiency and MRSA infection cannot be recommended.

## LIST OF ABBREVIATIONS

BS: = Bacteroides Spp
DFA: = Deep Femoral Artery
EC: = Escherichia Coli
EF: = Enterococcus Faecalis
EIA: = External Iliac Artery
FCA: = Femoral Circumflex Artery
GRA: = Girdlestone Resection-Arthroplasty
IPM: = Iliopsoas Muscle
LHBFM: = Long Head Biceps Femoris Muscle
MRI: = Magnetic Resonance Imaging
MRPA: = Multi-drug Resistent Pseudomonas Aeruginosa
MRSA: = Methicillin-Resistant Staphylococcus Aureus
PA: = Pseudomonas Aeruginosa
SA: = Staphylococcus Aureus
THA: = Total Hip Arthroplasty
VAC: = Negative-Pressure Vacuum Assisted
